# 5-methylcytosine turnover: Mechanisms and therapeutic implications in cancer

**DOI:** 10.3389/fmolb.2022.976862

**Published:** 2022-08-17

**Authors:** Marion Turpin, Gilles Salbert

**Affiliations:** ^1^ Sp@rte Team, UMR6290 CNRS, Institute of Genetics and Development of Rennes, Rennes, France; ^2^ University of Rennes 1, Rennes, France

**Keywords:** 5-Methylcytosine, cancer, DNA methylation, dynamics, TET, DNMT

## Abstract

DNA methylation at the fifth position of cytosine (5mC) is one of the most studied epigenetic mechanisms essential for the control of gene expression and for many other biological processes including genomic imprinting, X chromosome inactivation and genome stability. Over the last years, accumulating evidence suggest that DNA methylation is a highly dynamic mechanism driven by a balance between methylation by DNMTs and TET-mediated demethylation processes. However, one of the main challenges is to understand the dynamics underlying steady state DNA methylation levels. In this review article, we give an overview of the latest advances highlighting DNA methylation as a dynamic cycling process with a continuous turnover of cytosine modifications. We describe the cooperative actions of DNMT and TET enzymes which combine with many additional parameters including chromatin environment and protein partners to govern 5mC turnover. We also discuss how mathematical models can be used to address variable methylation levels during development and explain cell-type epigenetic heterogeneity locally but also at the genome scale. Finally, we review the therapeutic implications of these discoveries with the use of both epigenetic clocks as predictors and the development of epidrugs that target the DNA methylation/demethylation machinery. Together, these discoveries unveil with unprecedented detail how dynamic is DNA methylation during development, underlying the establishment of heterogeneous DNA methylation landscapes which could be altered in aging, diseases and cancer.

## Introduction

**FIGURE 1:**
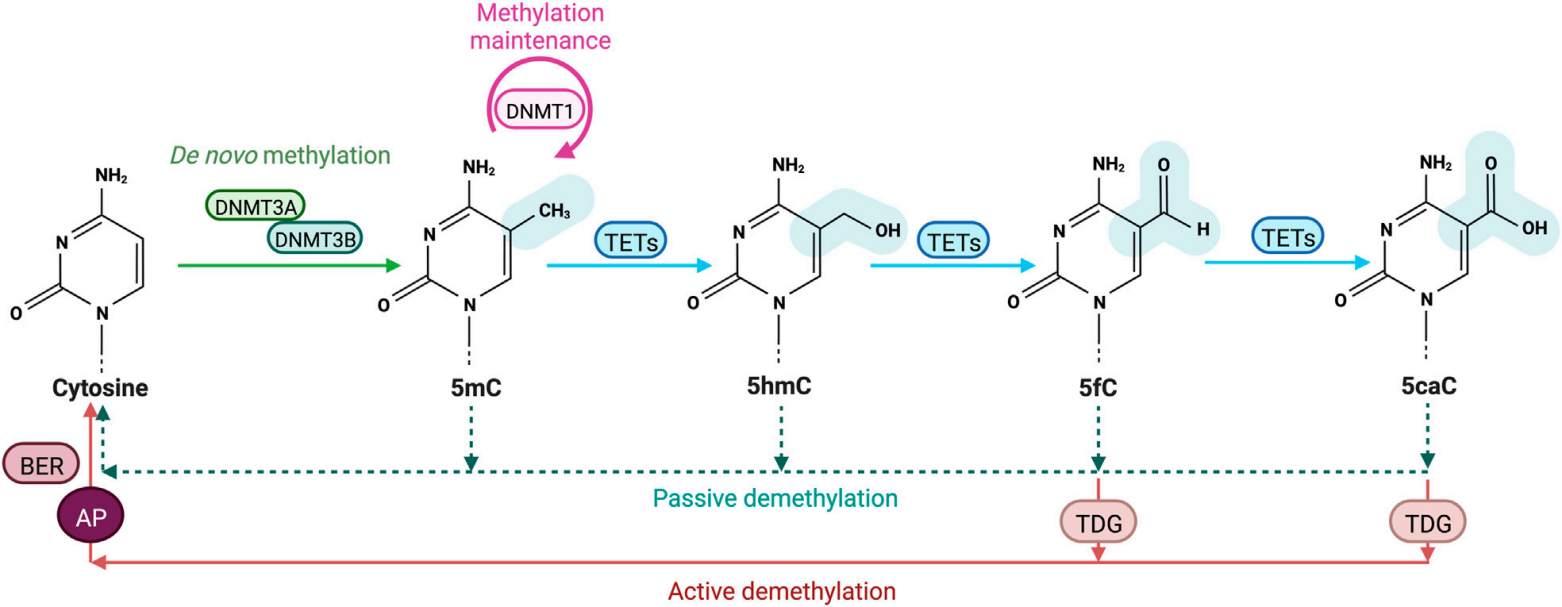
Cytosine methylation and oxidation cycle. In mammals, DNA methyltransferases (DNMTs) are responsible for the addition of a methyl group to the C5 position of cytosines to form 5-methylcytosines (5 mC). DNMT3A and DNMT3B drive *de novo* methylation during development, while DNMT1 is involved in maintaining DNA methylation by copying methylation patterns onto the newly replicated DNA strand. DNA methylation can be reversed in a passive way through replication-dependent dilution in absence of DNMT1. DNA demethylation can also occur actively, through Ten eleven translocation (TET) proteins that oxidize successively 5 mC into 5-hydroxymethylcytosine (5hmC), 5-formylcytosine (5 fC) and 5-carboxylcytosine (5caC) and the replacement of the oxidized bases 5 fC and 5caC by an unmodified cytosine through thymine DNA glycosylase (TDG)-mediated abasic site (AP) formation and base excision repair (BER).

One of the most well-characterized epigenetic modifications is DNA methylation, a mechanism involved in many biological processes including mammalian development, genomic imprinting, X chromosome inactivation, transposon silencing and gene regulation (Bird, 2002). In eukaryotes, DNA methylation is catalyzed by a family of enzymes called DNA methyltransferases (DNMTs) implicated in the covalent transfer of a methyl group from S’Adenosyl methionine (SAM) to the fifth carbon of a cytosine (C) pyrimidine ring to form 5-methyl cytosine (5mC) (Schübeler, 2015). Among DNMTs, the *de novo* methyltransferases DNMT3A and DNMT3B establish methylation patterns on unmethylated DNA through embryonic development (Figure 1) (Li and Zhang, 2014). These two enzymes with similar functions act with a regulatory enzyme called DNMT3L but intervene at different stages of embryogenesis (Kato et al., 2007). DNMT3B is activated in early embryos and during implantation whereas DNMT3A methylates DNA in the late stage of embryonic development, cell differentiation and in mature gametes (Smallwood et al., 2011). On the other hand, DNMT1 ensures methylation maintenance of the whole genome through cell divisions (Figure 1) and is responsible for the epigenetic inheritance of methylation patterns to somatic cells (Song et al., 2012). During the “S phase” of the cell cycle, DNMT1 is recruited to DNA by the nuclear protein ubiquitin-like plant homeodomain and RING finger domain 1 (UHRF1 also known as NP95), where it reproduces the methylation patterns of the parental DNA strand on the newly replicated DNA strand (Sharif et al., 2007).

DNA methylation is known to be an important regulator of gene expression and cell differentiation. Cytosine modifications especially occur at cytosines followed by a guanine nucleotide (CpGs) and most of CpGs (70–80%) are methylated genome-wide, including intergenic regions and gene bodies, likely preventing cryptic initiation of transcription in a cell-type specific fashion (Deaton and Bird, 2011). In mammals, CpGs are underrepresented and not uniformly distributed through the genome and some regions called CpG islands (CGIs) are enriched in CpGs. Usually, CpG poor regions are being methylated whereas CGIs are seemingly protected from DNA methylation. In somatic cells, almost 60% of CGIs are associated to gene promoters and transcription start sites (TSSs), mainly including housekeeping genes (Deaton and Bird, 2011). Therefore, effects of DNA methylation on gene transcription regulation are more likely location-dependent as well as cell-type specific. Indeed, 5 mC in gene bodies is positively associated to gene expression, and methylation of CGIs within or nearby promoters and TSSs is associated with transcriptional repression (Bird, 2002). Many studies suggested that gene repression may result from direct interference of DNA methylation with the binding of transcription factors (TFs) important for the transcriptional machinery. On the other hand, DNA methylation also has an indirect effect on the chromatin organization through the recruitment of methyl-CpG-binding proteins (MBPs) like MeCP2 that establishes dense chromatin environments through liquid-liquid phase separation (Deaton and Bird, 2011; Song et al., 2014).

Over the last years DNA (de)methylation has been widely investigated thanks to high throughput technological advances, thus allowing methylome profiling of a large panel of biological samples. Accumulating evidence suggest that DNA methylation is a highly dynamic mechanism driven by a balance between methylation and demethylation processes. However, although the distribution of DNA methylation in the genome is now well-documented at steady state, the dynamics of the underlying mechanisms requires more attention. In this review article, we will first give an overview of the latest advances highlighting DNA methylation as a dynamic cycling process with a constant turnover of cytosine modifications. We will also discuss how variable methylation levels could explain the epigenetic heterogeneity observed during early embryo development in mammals and might influence cellular differentiation.

## Mechanisms of DNA demethylation

For decades, DNA methylation was believed to be a stable epigenetic modification but recently several studies evidenced that it is rather dynamic than static. Indeed, 5 mC methylation is a reversible epigenetic modification counterbalanced by demethylation events (Yamagata et al., 2012). From a biological point of view, the simplest way to demethylate 5 mC would be to directly remove the methyl group from the cytosine residue. However, the carbon-carbon (C-C) bond of 5 mC is thermodynamically stable and demethylation mechanisms show variation among species. In flowering plants such as *Arabidopsis thaliana*, active replacement of 5mCs by unmodified cytosines is initiated by the glycosylase activity of Demeter (DME)/repressor of silencing 1 (ROS1) enzymes from the base excision repair (BER) machinery (Zhu, 2009). In mammals, however, no ortholog of DME/ROS1 has been identified yet. Previous work from Bhattacharya et al. have suggested that methyl-CpG-binding domain protein 2 (MBD2) is able to catalyze the excision of the methyl group, leading to methanol release (Bhattacharya et al., 1999). This hypothesis has been largely contested as the protein lacks any enzymatic domain and the results were not reproduced by other laboratories. Nonetheless, methyl groups from 5 mC modifications can be erased by many other different mechanisms which can be passive or active (Wu and Zhang, 2010). On one hand, passive demethylation refers to the dilution of 5 mC marks through successive cell divisions following replication in absence of remethylation or methylation maintenance by DNMT1. Indeed, a reduction in DNMT1 activity leads to a progressive decrease in methylation level through cell divisions. On the other hand, active DNA demethylation corresponds to replication-independent processes where methylated cytosines are removed and replaced by unmodified cytosines through enzymatic modifications (Wu and Zhang, 2017). In mammals, potential candidates have been suggested to achieve DNA demethylation such as the nuclear protein growth arrest- and DNA damage-inducible 45 (GADD45) proteins (Barreto et al., 2007), the Elongator complex protein 3 (ELP3) (Wu and Zhang, 2010) but experimental evidence for their direct involvement in DNA demethylation in cells is still lacking.

Transformation of 5 mC to T by DNMT3A/B-enhanced deamination has been proposed to trigger recruitment of the T:G mismatch DNA glycosylase (TDG) and the BER machinery during estrogen receptor-mediated transcription activation (Métivier et al., 2008). However, 5 mC deamination is inhibited by SAM and such a mechanism is unlikely to take place in cells. Additional breakthrough came with the simultaneous discovery of 5-hydroxymethylcytosine (5hmC) and Ten eleven translocation (TET) proteins that oxidize 5 mC into 5hmC using reduced iron (Fe2+) and α-ketoglutarate (α-KG) as cofactors (Figure 1) (Tahiliani et al., 2009). The 5hmC base can be further oxidized into 5-formylcytosine (5 fC) and 5-carboxylcytosine (5caC) (Tahiliani et al., 2009; Ito et al., 2011; Wu and Zhang, 2017) which are further converted into unmodified cytosines through the action of TDG and the DNA repair BER machinery (Figure 1). Alternative mechanisms include 5mC and 5hmC deamination into Thymine or 5-hydroxymethyluracil (5hmU) by activation-induced cytidine deaminase/apolipo-protein B mRNA-editing enzyme complex (AID/APOBEC) (Cortellino et al., 2011). The resulting T/G mismatches may further be targeted by DNA glycosylases such as TDG and methyl-binding domain protein 4 (MBD4) from the BER pathway (Morgan et al., 2004). However, recent data indicate that the majority of 5hmU in the genome originates from oxidation of Ts by TETs (Pfaffeneder et al., 2014). In addition, oxidized forms of 5 mC are poor substrates for methylation maintenance by DNMT1 and as such lead to a lower fidelity in 5mc patterns after DNA replication. Finally, TDG-independent removal of 5 fC and 5caC has been suggested to occur through direct deformylation or decarboxylation in cells but the involved enzymes still need to be discovered (Iwan et al., 2018; Kamińska et al., 2021).

## Interplay between active DNA demethylation and passive demethylation

DNMT1 is responsible for the maintenance of DNA methylation patterns during DNA replication. The multifunctional protein UHRF1 has been reported to act as an important cofactor of DNMT1 in DNA maintenance methylation (Sharif et al., 2007). UHRF1 is a multi-domain containing protein regulating epigenetic modifications and acting as a mediator between histone modifications and DNA methylation. UHRF1 ensures DNA maintenance methylation through its central SET- and RING-associated (SRA) and C-terminal really interesting new gene (RING) domains. UHRF1 specifically recognizes hemi-methylated DNA at replication forks and flips methylated cytosines via its SRA domain. This base-flipping mechanism recruits DNMT1 to its target sites on the newly synthetized DNA strand during S phase and allows the exposure of the unmodified cytosine to DNMT1 (Berkyurek et al., 2014; Bianchi and Zangi, 2014; Qin et al., 2015). The ubiquitin E3 ligase activity of UHRF1 RING domain in ubiquitinating lysines 23 and 18 of histone H3 (H3K18Ub, H3K23Ub) at the N terminus of PCNA-associated factor 15 (PAF15) is also essential for promoting DNMT1 binding to DNA replication sites (Nishiyama et al., 2013; Qin et al., 2015). UHRF1 also increases DNMT1 activity and specificity through a direct interaction between its SRA domain and replication focus targeting sequence (RFTS) domain of DNMT1 (Bashtrykov et al., 2014; Berkyurek et al., 2014). It has been recently reported that hemi-methylated DNA induces a conformational opening of UHRF1 facilitating histone recognition (Fang et al., 2016). Indeed, UHRF1 is implicated in heterochromatin formation, thus keeping a repressive chromatin landscape. By tandem Tudor domain (TTD) and a plant homeodomain (PHD) domains, UHRF1 recognizes histone H3 unmodified arginine 2 (H3R2) and transcriptionally repressive chromatin marks H3K9me2/3 on chromatin (Rajakumara et al., 2011; Cheng et al., 2013). These histone marks are necessary for H3K18 and H3K23 ubiquitination and may contribute to a cooperative interplay between histone modifications by UHRF1 and DNA methylation. UHRF1 SRA domain has also been reported to recruit the histone deacetylase 1 (HDAC1) associated with chromatin compaction and stabilizing DNMT1 activity (Unoki et al., 2004). DNMT1 directly interacts via its PCNA-binding domain (PBD) domain with PCNA, the processivity factor of the DNA replication machinery.

Impairment of DNA maintenance methylation reduces global DNA methylation and results in passive demethylation characterized by a progressive dilution and loss of 5 mC marks during successive DNA replication cycles. Several processes can be involved in replication-dependent passive DNA demethylation including the downregulation of DNMT1/UHRF1 complex (Oda et al., 2013), the impairment of DNMT recruitment on DNA (Oda et al., 2013), a decrease in the levels of SAM substrate (Ulrey et al., 2005), and a reduced enzymatic activity (Seiler et al., 2018). Indeed, replication-dependent passive DNA demethylation can be promoted by oxidized methylcytosines 5hmC, 5 fC and 5caC, together called oxi-mCs. Indeed, the DNMT1/UHRF1 complex recognizes 5 mC at hemi-methylated CpGs during S phase and methylates the unmodified cytosine on the newly replicated DNA strand but does not recognize hemi-modified CpGs with oxi-mCs resulting from TET activity, thus preventing the reestablishment of symmetrical CpG methylation on the newly synthetized strand (Otani et al., 2013). Therefore, this may lead to a progressive dilution of 5mCs in daughter cells due to a reduced DNMT1/UHRF1 enzymatic activity through successive cell divisions. For instance, it has been shown in mouse zygotes that paternal demethylation involves 5 mC oxidation by TETs into 5hmC, 5 fC and 5caC followed by a replication-dependent dilution 5 fC and 5caC during mouse preimplantation development (Inoue et al., 2011). Seiler et al., also recently reported that the presence of oxi-mCs would prevent the formation of DNMT1/UHRF1 complex, resulting in a decrease in maintenance methylation activity (Seiler et al., 2018). Therefore, TET-mediated oxidation of 5 mC can lead to passive DNA demethylation.

## DNA (de)methylation dynamics through embryonic development

DNA methylation patterns are essential for cell fate decisions during mammalian development and act as epigenetic barriers by limiting the developmental potential of cells and restricting their differentiation to avoid a regression into an undifferentiated state. Although DNA methylation landscapes are stably maintained in somatic cells, the genome undergoes a dynamic two-step genome wide reprogramming (Bird, 2002). First, a cycle of demethylation and remethylation occurs in PGCs during germ cell development where the parental imprints are erased and re-initiated in the developing gametes to further regain pluripotency (Lee et al., 2014). After fertilization, a second wave of reprogramming takes place in pre-implantation embryos in which sex specific methylation patterns are established whereas imprinted marks are maintained (Seisenberger et al., 2012).

Normal development in mammals is tightly conditioned by the regulation of *de novo* methylation and demethylation, and the functions of the regulators have been largely studied using mouse and human embryonic stem cells (mESCs and hESCs) as powerful model systems for both *in vivo* and *in vitro* assays. Both mESCs and hESCs derive from the inner cell mass of blastocyst-stage pre-implantation embryos but hESCs reflect a later phase in development than mESCs in addition to having distinct biological properties (Verma et al., 2018). In hESCS, the DNMT1, DNMT3A and DNMT3B enzymes are expressed along with TET1, TET2 and TET3 whereas TET3 is usually absent from mESCs. The roles of DNMTs and TET proteins during early embryo development have been characterized in both mouse and human using conventional gene targeting methods and genome-editing tools such as clustered regularly at interspaced short palindromic repeats (CRISPR) and CRISPR associated (Cas) nucleases (Dawlaty et al., 2013; Liao et al., 2015).

### DNMT and TET proteins are essential for normal development

DNA (de)methylation dynamics plays a key role in the spatio-temporal regulation of gene expression, the maintenance of genome stability and embryonic development (Cartron et al., 2015). The deletion of TETs and DNMTs results in global changes of DNA methylation patterns in mESCs and single knockouts (KO) induces embryonic (DNMT1, DNMT3B) or early postnatal (DNMT3A, TET3) lethality in mice (Li et al., 1993; Lei et al., 1996; Okano et al., 1999; Gu et al., 2011; Liao et al., 2015). The effects of TET genes loss on DNA methylation in early embryonic development have been well-studied and single or combined depletion of TET1 and TET2 (DKO) in mESCs revealed a reduction of 5hmC levels, and a skewed differentiation without affecting cells pluripotency (Dawlaty et al., 2013, 2011). Knockout mouse models with a double TET1/2 KO are still viable and fertile but the embryonic development is impaired with many mid-gestation developmental abnormalities (Dawlaty et al., 2013; Hon et al., 2014). These observations indicate that the deficiency of one TET member does not interfere with embryogenesis and cell pluripotency but rather influence differentiation and lineage commitment. Consistently, the inactivation of all TET proteins by triple knockout (TKO) impairs cells differentiation and causes locus-specific hypermethylation of enhancers and gene promoters known to be involved in development (Dawlaty et al., 2014; Verma et al., 2018; Ginno et al., 2020). In line with these data in mouse embryos and mESCs, the same results have been obtained in TKO hESCS despite their higher CpG methylation levels than mESCs, suggesting that TET TKO is not compatible with embryogenesis (Von Meyenn et al., 2016a). Altogether, loss of function experiments demonstrated the importance of TET enzymes in cell differentiation during development and similarly, several studies also analyzed the impact of the removal of DNMTs through development.

DNMT1 disruption in mESCs has been found to result in an overall demethylation but the null mutant cells remain viable with a normal proliferation (Li et al., 1993; Lei et al., 1996). On the opposite, DNMT1 KO is lethal in dividing somatic mESCs as well as for hESCs lacking DNMT1 undergoing rapid cell death (Liao et al., 2015). Moreover, the deletion of DNMT1 in mouse embryos have been showed to cause embryonic lethality past midgestation at around 9.5 days post coitum (dpc) (Liao et al., 2015). Although mice with DNMT3A and DNMT3B heterozygous deletions are so far viable and fertile, single DNMT3A homozygous mutant embryos develop to term but most of them die within 4 weeks after birth, whereas mice with a single DNMT3B KO show several developmental abnormalities (Okano et al., 1999; Liao et al., 2015). Conversely, single and combined DNMT3A and DNMT3B mutant hESCs remain pluripotent while progressively loosing DNA methylation over cell cycles (Liao et al., 2015). Previous studies also reported the loss of hESCs capacity to generate teratomas at early culture passage numbers (Okano et al., 1999; Chen et al., 2003). Likewise, a genome wide decrease in *de novo* methylation during development is also observed in DNMT3s DKO mESCs as well as in mouse early embryos deficient in both of DNMT3A and DNMT3B, which leads to embryonic lethality (Okano et al., 1999; Chen et al., 2003; Kinoshita et al., 2021). Furthermore, a triple knockout (TKO) of all DNMTs has been performed in mESCs by gene targeting and without any surprise a lack of methylation was observed as well as a conservation of stem cell properties and self-renewal (Tsumura et al., 2006). Nevertheless, hESCs lacking all three DNMTs did arrest rapidly in G1 phase of the cell cycle, thus leading to cell death (Liao et al., 2015). The effect of DNMT TKO has been further investigated *in vivo* in mouse embryos by transferring nuclei from DNMT TKO mESCs into enucleated oocytes which were subsequently inserted into a pseudo pregnant female (Sakaue et al., 2010). The ensuing mouse embryos were found to develop normally into blastocysts in absence of DNA methylation but some growth and lineage abnormalities were observed in specific lineages such as epiblast lineage differentiation (Sakaue et al., 2010). Taken together, these findings highlight the importance of DNMTs mediated DNA methylation and demethylation by TETs during embryonic development.

### DNA methylation turnover is driven by a balance between DNMTs and TETs

**FIGURE 2:**
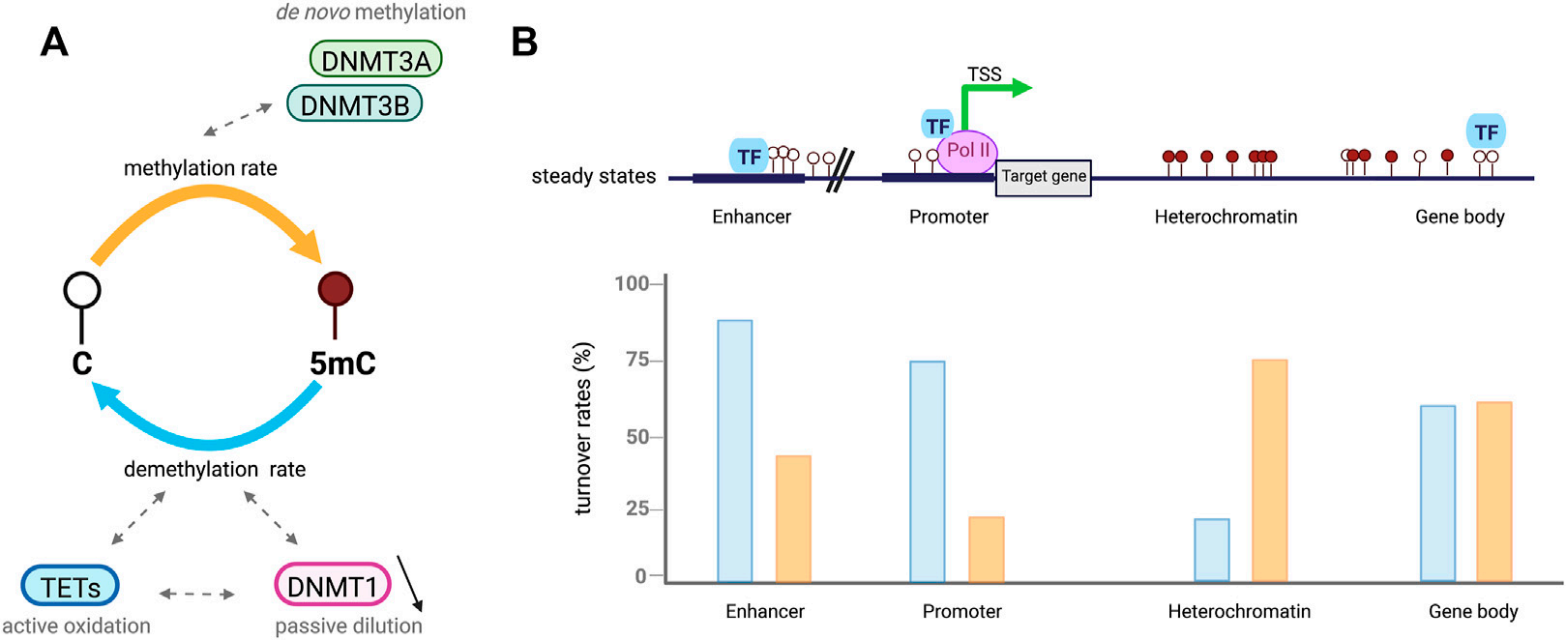
Cytosines undergo dynamic methylation turnover. **(A)**. In a cell population, steady-state DNA methylation is reached when overall DNA methylation rates (orange) are equivalent to demethylation ones (blue). Given an identical steady-state, CpGs are influenced by different methylation (*de novo*) and demethylation (active oxidation and passive dilution) rate combinations called DNA methylation turnover. **(B)**. Example of DNA methylation turnover depending on CpGs location in the genome, based on Ginno et al. (2020). In euchromatic context, CpGs at regulatory regions including enhancers and promoters present low steady-state DNA methylation levels associated with increased demethylation rates (blue) compared to methylation ones (orange). High rates of passive demethylation are observed at promoters while active demethylation is more important at enhancers. CpGs in heterochromatic regions have high steady-state methylation which tends to be associated with low DNA methylation turnover. However, CpGs located at gene bodies are also highly methylated but tend to have a higher turnover.

DNA methylation is tightly controlled by DNMTs and TETs which are, despite their opposing activities, both expressed during ESCs differentiation into somatic lineages. This co-expression leads to genome-scale oscillatory dynamics and cell-to-cell heterogeneity further impacting gene expression (Gu et al., 2018; Rulands et al., 2018; De Riso et al., 2020). TET activity has been found to require DNMT3 expression to keep steady-state DNA methylation levels in the genome of pluripotent ESCs (Charlton et al., 2020). DNA methylation steady state is defined as the equilibrium of DNA methylation levels reached when the average methylation rates are similar to demethylation ones (Figure 2A) (Ginno et al., 2020). The steady state is itself characterized by DNA methylation turnover referred as different combinations of enzymatic rates of methylation/demethylation, suggesting that CpGs with a similar steady state can vary from each other by having distinct methylation/demethylation rate values (De Riso et al., 2020; Ginno et al., 2020). DNA methylation turnover is very active through cell fate transition and the high oscillatory methylation dynamic observed appears to be context-specific in the genome (Figure 2B). Paradoxically, the two antagonizing enzyme families target overlapping genomic regions including large undermethylated regions so-called canyons, TSSs and gene distal regulatory elements such as enhancers (Gu et al., 2018; Luo et al., 2018; Rulands et al., 2018; Song et al., 2019; Ginno et al., 2020). In fact, TETs are locally recruited at differentially methylated regions (DMRs) overlapping promoters and active somatic enhancers where they maintain a hypomethylated state (Figure 2B). Although those regulatory regions are highly methylated in pluripotent cells, TET proteins remain highly expressed but if DNMT3 is lost a rapid TET mediated demethylation occurs (Charlton et al., 2020). These results extended previous hypotheses stating that DNA methylation is a dynamic process where cytosines are constantly methylated/demethylated, associated with a dynamic turnover of DNMTs and TETs (Lee et al., 2014). In addition, Gu *et al* also recently reported in mESCs that DNMT3A and TET1 cooperate to control gene expression around promoters and canyons (Gu et al., 2018). Indeed, TET1 proteins are able to limit the binding of *de novo* DNA methyltransferases to regulatory elements of the genome whereas DNMT3A can conversely restrain TET1 engagement at CGIs. These observations are in line with previous studies indicating that TET association with promoter-CGIs and canyons keep DNA methylation at low levels and protect those regions from being hypermethylated (Peat et al., 2014; Wiehle et al., 2016). For cells that are cycling, the presence of TET-mediated oxidation marks on one strand of CpG dinucleotides can promote passive DNA demethylation by preventing the methylation of the CpG on the opposite strand by DNMT1 after DNA replication, (DeNizio et al., 2021). Recent work in mESCs also demonstrated the synergistic actions between members of each family of methylating and demethylating enzymes with 5 mC oxidation marks promoting passive demethylation and DNMT1 controlling *de novo* methylation by DNMT3s during development. Unmodified DNA can be remethylated by DNMT3 enzymes during the next G1 phase of the cell cycle but newly synthetized dsDNA can no more be methylated by DNMT3s through the S phase when DNMT1 is lacking (Ito et al., 2022). This suggests that DNMT1 has an important role in the regulation of DNA methylation by DNMT3s, which could be either an enzymatic dependent regulation by direct interaction or an indirect binding to a protein partner common to both DNMT subgroups.

The DNA methylome is shaped by a local competition between DNA methylation by DNMTs and demethylation by TET enzymes, depending on their recruitment and activity towards CpGs. DNMTS and TETs do not bind DNA randomly but rather target some CpG sites influenced locally by adjacent nucleotides and the CpG density of flanking sequences (Adam et al., 2020; Mallona et al., 2021). Although DNMTs and TETs usually target cytosines within a CpG environment, DNA methylation can also occur at cytosines preceding other bases than G so-called non-CpG methylation or mCH, where H equals to A, C, or T (Lister et al., 2013; He and Ecker, 2015). Abundant levels of mCH are found in pluripotent stem cells, oocytes, glial cells and neurons whereas it is rare in adult differentiated cells (Lister et al., 2013). DNMTs methylate CpGs with different efficiencies depending on neighboring sequences impacting CpG recognition, thus influencing 5 mC levels in the genome (Adam et al., 2022). As for DNMTs, the recognition of a target locus by TET enzymes is also impacted by surrounding DNA bases. Although this idea has been somewhat controversial, recent studies demonstrated that the catalytic domain of TETs has a sequence specificity with a higher preference for some motifs in the genome (Hu et al., 2015; Ravichandran et al., 2021). TET1 and TET3 have been shown to rapidly oxidize methylated CpG sites (mCpGs) with an A or a T at the -1 and +1 positions respectively. On the other hand, TET2 has a strong preference for mCpGs and to a lesser extent for mCpAs with a T at the +1 position as well as mCpCs with a at the +1 base (DeNizio et al., 2021). However, similar affinities have been found for mCpA and mCpC contexts when there is a G at the +1 site.

### DNA methylation dynamics is regulated by the synergistic actions of multiple actors

Beyond the cooperation between TETs and DNMTs in regulating DNA methylation dynamics, many additional factors including chromatin modifications, protein partners like TFs and non-coding RNAs combine to govern DNA methylation turnover.

**FIGURE 3:**
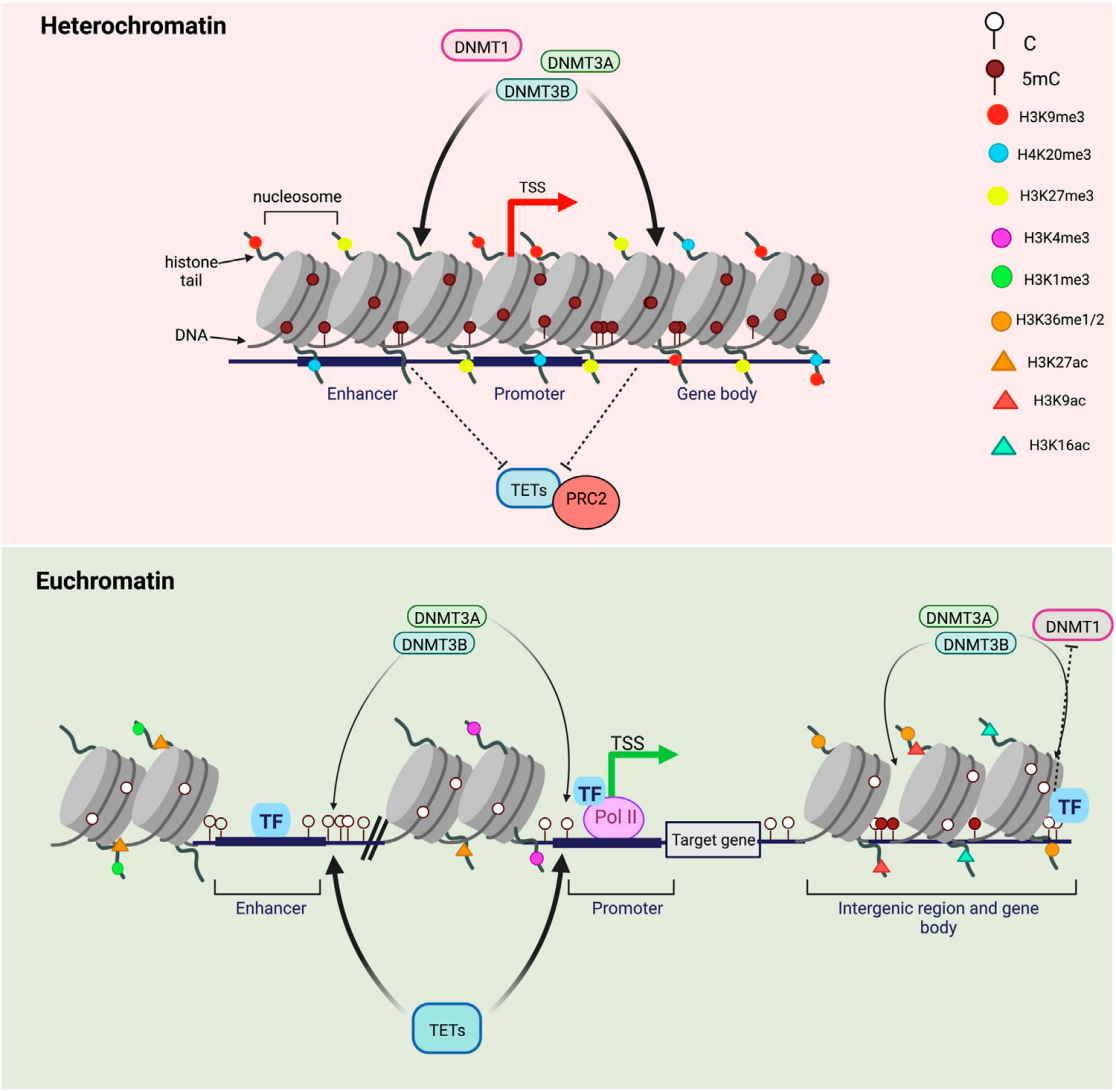
DNA methylation turnover in heterochromatin versus euchromatin. Transcriptionally silent genes are found in highly-condensed chromatin regions called heterochromatin and marked by repressive histone marks H3K9me3, H3K27me3 and H4K20me3. DNA methylation levels in heterochromatin are high and DNA methylation turnover rates are usually low. CpGs in heterochromatic regions are highly methylated by DNMTs and inaccessible by TET proteins. On the opposite, transcriptionally active genes are associated with a less-condensed, nucleosome-depleted and accessible chromatin known as euchromatin. In transcriptionally active regions, gene promoters are enriched in H3K4me3 and H3K27ac, enhancers in H3K4me1 and H3K27ac and gene bodies in H3K9Ac and H3K36me. Highly methylated CpGs within highly transcribed genes, gene bodies and nearby regulatory regions (enhancers and promoters) are subjected to high methylation turnover rates while hypermethylated CpGs within intergenic regions have lower TET and DNMT engagement. Transcription factors binding can regulate both methylation by DNMTs and demethylation by TETs.

In the nucleus of eukaryotic cells, genomic DNA is packaged around histone proteins (H1, H2. A, H2.B, H3, and H4) to form the fundamental unit of chromatin: the nucleosome (Wolffe, 1998). Each nucleosome is composed of core histones (two dimers of H2. A/H2.B and one tetramer of H3/H4) around which DNA is wound. Many epigenetics marks including post-translational histone modifications (PTMs) regulate gene expression by impacting the chromatin structure as well as inducing nucleosome repositioning and chromatin remodeling (Fenley et al., 2018). DNA accessibility to DNA-binding proteins is therefore altered by the presence of PTMs impacting the chromatin organization through development. Chromatin is divided into different states depending on PTMs: transcriptionally active genes are associated with a less-condensed, nucleosome-depleted and accessible chromatin known as euchromatin while transcriptionally silent genes are linked to highly-condensed and inaccessible chromatin regions called heterochromatin (Tamaru, 2010). DNA methylation turnover rates have been found to be opposite between euchromatin and heterochromatin which has lower turnover rates associated with high DNA methylation levels (Figure 3) (Ginno et al., 2020). Inactive heterochromatic regions are usually marked by repressive histone marks including the trimethylation of lysine nine on histone H3 (H3K9me3), H3K27me3 and H4K20me3 (Stachecka et al., 2021). In euchromatic transcriptionally active regions, poised or active genes promoters are enriched for trimethylation of lysine four on histone H3 (H3K4me3) while active enhancers are marked by the monomethylation H3K4 and the acetylation of histone H3 on K27 (H3K27ac) (Figure 3) (Calo and Wysocka, 2013). Active histone marks are also found at gene bodies such as the acetylation of histone H3 (H3K9Ac) and H4 (H4K16Ac) as well as the histone H3 monomethylation on K36 (H3K36me) (Price et al., 2019). Recently, Ginno et al. discovered that, when located within highly transcribed genes, gene bodies and nearby regulatory regions, highly methylated CpGs undergo high methylation turnover rates (Figure 2) (Ginno et al., 2020). Surprisingly, CpGs outside regulatory regions associated with repressive chromatin marks have a high DNA methylation steady state despite extremely varying enzymatic levels. Conversely, hypermethylated CpGs within intergenic regions show lower TETs and DNMTs rates (Ginno et al., 2020). DNA methylation is also believed to be interconnected with histone modifications as TET1 and DNMT3A alter histone landscapes at the regions of the genome they bind. For instance, these two competitive enzymes modulate the histone mark H3K27me3 by the Polycomb repressive complex 2 (PRC2), an important mark of gene silencing during early development and tumorigenesis (Figure 3) (Gu et al., 2018). Moreover, nucleosome occupancy has been shown to influence DNA methylation turnover at regulatory regions and inhibit DNMT1 and DNMT3 activities (Ginno et al., 2020).

Dynamic DNA methylation landscapes are shaped by a local competition between TETs and DNMTs for CGI association as well as other proteins partners such as TFs. Actually, DNA methylation and TFs binding act in a synergistic manner to regulate the spatio temporal expression of genes and chromatin remodeling, leading to local DNA methylation changes. Zinc finger cysteine-X-X-cysteine (ZF-CXXC) domain-containing proteins specifically target unmethylated CpGs at CGIs which are commonly hypomethylated and where they have chromatin-modifying activities (Gu et al., 2018; Van De Lagemaat et al., 2018; De Riso et al., 2020). The first CXXC domain containing protein to have been discovered in mammals a few years ago is the CXXC finger protein 1 (CPF1), a key component of the SETD1 (SET domain 1) H3K4 methyltransferase complex responsible for H3K4 methylation (Clouaire et al., 2012). CFP1 is known to be a key regulator of H3K4 methylation at CGIs associated with TSSs and promoters of active genes mostly, therefore influencing gene expression (Brown et al., 2017). Until now, a dozen of ZF-CXXC containing proteins have been identified in humans and classified into different subgroups according to their chromatin-modifying activities (Long et al., 2013): CFP1 and mixed lineage leukaemia proteins 1/2 (MLL1 and MLL2) are associated with H3K4 methylation, H3K36me1/2 methylation is controlled by lysine-specific demethylases 2A and 2B (KDM2A and KDM3B), DNMT1 drives DNA methylation while TET1 and TET3 regulate DNA hydroxymethylation through their CXXC domain (Tahiliani et al., 2009; Gu et al., 2011; Vacík et al., 2018). By recognizing and binding to non-methylated CGIs independently of the sequence, CXXC proteins compete with DNMTs and restrain DNA methylation. This protection helps to keep unmethylated regions accessible and transcriptionally active (Long et al., 2013). Conversely, DNA methylation equilibrium can be preserved thanks to proteins such as MDB family proteins that bind methylated CpGs often located at CGI gene promoters to maintain DNA methylation and transcriptional silencing through interactions with chromatin remodelers (Du et al., 2015). For instance, in human kidney cancer cells devoid of the protein Kaiso, the genome is widely hypermethylated whereas some genomic regions like promoters and enhancers are protected from demethylation (Kaplun et al., 2021). Besides being a MBD, Kaiso may control DNA methylation equilibrium by attracting DNMT3A/B to neighboring regions and protecting pluripotent factors binding sites (like Oct4 and Nanog), enhancers and super enhancers from hypermethylation. Kaiso also competes at methylated binding sites with Kruppel-like factor 4 (KLF4), implicated in the TET2-mediated demethylation of enhancers and super-enhancers during somatic cells reprogramming (Sardina et al., 2018; Kaplun et al., 2021). The overall genome methylation levels are further impacted by the downregulation of TET1 transcription in Kaiso deficient cells, leading to global DNA hypermethylation. DNA methylation by DNMTs and TET-mediated demethylation can be promoted by TF binding to unmethylated and methylated regions of the genome respectively (Rasmussen and Helin, 2016). Indeed, some TFS have interactions with DNMTs and TET enzymes or both and can attract these proteins to particular genomic sequences. For example, promyelocytic leukemia/retinoic acid receptor alpha (PML–RAR) protein recruit DNMT1 to the retinoic acid receptor beta (RARB) gene promoter where transcriptional repression is held (Di Croce et al., 2002). On the opposite, RUNX1 is an important TF for hematopoietic development known to recruit the DNA demethylation machinery in hematopoietic cells and induce gene activation (Suzuki et al., 2017).

Emerging evidence suggest the existence of long non-coding RNA (lncRNA)-mediated DNA methylation. LncRNAs are transcripts with a length over 200 nucleotides which have been shown to interact with DNMTs and TETs through different mechanisms (Huang et al., 2022). Methylating and demethylating enzymes can be directly recruited to locus-specific target gene promoters by lncRNAs, thus modulating DNA methylation patterns. LncRNAs can also indirectly recruit DNMTs by communicating with Enhancer of Zeste homolog 2/Prohibitin 2 (EZH2/PHB2) and induce a repressive chromatin state whereas GADD45A mediated recruitment of TETs to CGI promoters by lncRNAs often leads to chromatin opening and gene activation (Su et al., 2018; Arab et al., 2019). Moreover, lncRNAs can control DNMTs and TETs expression and activities which could impact DNA methylation dynamics (Yang Z. et al., 2021).

## Mathematical modelling to study DNA methylation dynamics

Over the past few years, many mathematical models and quantitative analysis of DNA methylation profiles have enabled to deepen knowledge on DNA methylation dynamics at differentially methylated regions (DMRs) involved in establishing cell-type specific heterogeneity. A classical model of methylation dynamics has been first proposed by Otto and Walbot (Otto and Walbot, 1990) and Pfeifer et al. (Pfeifer et al., 1990), describing a global DNA methylation equilibrium reached in dividing cells upon interactions between both *de novo* and maintenance DNMTs. These models were then further improved by Genereux et al. (Genereux et al., 2005) and assuming that *de novo* methylation of a CpG dyad can be asymmetric between parental and daughter strands of individual DNA molecules. Based on previous hairpin-bisulfite PCR analyses of fully methylated, hemimethylated, and unmethylated CpG dyads (Laird et al., 2004), they estimated the methylation rates of these three states within the promoter of the human gene FMR1 by using maximum likelyhood. The classical model has been also adapted by Sontag et al. to a more global methylation dynamics, based on DNMT cooperativity (Sontag et al., 2006). By using a Markov chain approach with a defined steady-state equilibrium including DNA strands asymmetry, they confirmed the two metastable states of hypermethylation and hypomethylation in somatic cells (Sontag et al., 2006). Numerous other models were subsequently developed by integrating new parameters in order to specify methylation kinetics and precise DNA methylation turnover, and by considering active DNA demethylation by TETS proteins involved in 5 mC hydroxymethylation as well as the existence of non CpG methylation. Such theoretical and *in vitro* works in cultured ESCs revealed many properties of the enzymes responsible for these variations in methylation levels (Jeltsch and Jurkowska, 2014; Von Meyenn et al., 2016b).

The developed mathematical models explained the dynamics underlying steady state DNA methylation levels. For years, bulk cell measurements approaches used the average DNA methylation level of a cell population to define the methylation state of CpGs without considering that each CpG may exist in a dual state (methylated or unmethylated). The development of high-resolution single-cell approaches and especially single cell whole genome Bisulfite Sequencing (scBS-Seq) largely contributed to unravel DNA methylation variations in differentiating cells, at the genome scale. Using whole genome scBS-Seq, several studies demonstrated the emergence of methylation heterogeneity in mESCs(Guo et al., 2015; Rulands et al., 2018). However, these methods, even at the single-cell level, were limited in inferring DNA methylation and demethylation rates from average methylation levels for individual CpGs in heterogeneous cell populations and tissues.

To get through the challenges faced by bulk measurements and sequence dependent approaches, novel experimental methods combining single-cell sequencing, modeling, and knock-out of (de)methylation enzymes revealed highly dynamic methylation changes in early development both *in vitro* and *in vivo* (Rulands et al., 2018; Song et al., 2019; Charlton et al., 2020; De Riso et al., 2020; Shi et al., 2021). The co-engagement of DNMTs and TET enzymes on the same DNA molecule has been evidenced not long ago by Rulands et al. by using single-cell sequencing and a modelling method. This co-occurrence of DNMT3 and TETs leads to oscillatory methylation dynamics among cells during early embryogenesis (Rulands et al., 2018). By considering cell-to-cell heterogeneity, the mathematical model developed by De Riso et al., provided evidence for the simultaneous actions of DNA methylation and demethylation processes in some specific regions near gene promoters in individual cells. The results suggest that these cell-specific mechanisms are influenced by neighboring CpGs while they seem to reach a steady-state equilibrium in the cell population as a whole (De Riso et al., 2020).

DNA methylation dynamics has been found to depend on the co-expression of DNMT3 and TETs but also on TF binding, creating local hypomethylation that could be advantageous for additional TF engagement and resulting in DNA methylation heterogeneity locally but also at the genome scale (Charlton et al., 2020; Ginno et al., 2020). For each single CpG in the genome, DNA methylation and demethylation rates can be inferred by a dynamical model for DNA methylation associated with a statistical error model. DNA methylation heterogeneity in a cell can be determined by a DNA methylation state transition model called Methyltransition (Zhao et al., 2020). This model is based on the hypothesis that the transition of DNA methylation state of a CpG over a single cell cycle happens in three steps: 1) DNA is passively demethylated through replication, 2) DNA methylation transitions are mediated by TETs and DNMTs, and 3) DNA methylation transition combination states are merged from both homologous chromosomes. Consequently, a methylation state ratio vector has been created to describe the global DNA methylation states for a given single cell. To link the two methylation states before and after a cell cycle, a transition matrix was generated and involves the three parameters which represent the probabilities of DNA methylation maintenance, active demethylation, and *de novo* methylation (Zhao et al., 2020). Methyltransition revealed a programmed epigenetic heterogeneity in early embryos which is defined by the first DNA methylation status at the zygote phase in development (Zhao et al., 2020). Recently, a new quantitative measure of DNA methylation known as the methylation occurrence ratio has been developed to study the interplay between DNA methylation and transcription regulation (Shi et al., 2021). This metric highlighted the antagonizing activities of TETs and DNMTs, especially near TSS-proximal regions. Compared with other conventional metrics such as average methylation and the methylation variation, methylation occurrence is able to detect larger sets of regulatory regions and appears to be a better predictor of gene expression (Shi et al., 2021).

These results demonstrate that DNA methylation can be modelized as a highly dynamic and context-specific process occurring mostly at DMRs, including enhancer regions characterized by oscillations in DNA methylation levels. Taken together these findings unveil with unprecedented detail how DNA methylation dynamics during development underlies the establishment of heterogeneous DNA methylation landscapes which could be altered in aging, diseases and cancer.

## Therapeutic implications

Aberrant DNA methylation is a hallmark of cancer and is characterized by a genome-wide hypomethylation as well as a global genomic instability. Concomitant with global hypomethylation, hypermethylation of CGIs next to TSSs from tumor suppressor genes (TSGs) resulting in their silencing as well as proto-oncogenes overexpression are also frequently observed. Aberrant DNA methylation leads to the formation and/or progression of cancer including hematological malignancies and solid tumors (Kulis and Esteller, 2010) and methylation defects are frequently associated with a loss of activity of TET enzymes (Zhang Y. W. et al., 2017).

Within the last years, based on the reversible nature of epigenetic marks, small molecules referred to as epidrugs were developed to target epigenetic regulators (Fardi et al., 2018). Aberrant methylation can be therapeutically removed by epidrugs (Table 1) including 5-azacytidine (5-AZA) and 5-aza-2′-deoxycytidine (decitabine), inhibiting DNMTs and inducing a reprogramming of the genome. These Food and Drug Administration (FDA)-approved inhibitors of DNMTs are usually indicated for the treatment of solid tumors and hematological malignancies including myelodysplastic syndromes (MDS) and acute myeloblastic leukemia (AML) in adults (Bohl et al., 2018; Kazachenka et al., 2019). These nucleoside analogs are incorporated into DNA in place of cytidine during replication and covalently trap DNMTs in an irreversible complex which is followed by degradation of the methyltransferasees by the proteasome (Mack, 2006). Such DNMT inhibition induces a global hypomethylation of the genome and restores the expression of aberrantly repressed genes such as TSGs. These epidrugs have been tested for various cancers and several clinical trials have been performed on patients with solid tumors (Kazachenka et al., 2019). Data indicated reliable anti-tumor effects but although it reactivates silenced genes at low doses, it shows high cytotoxicity at higher concentrations. In addition, the migratory and invasive capacities of tumor cells have been observed to be also increased by decitabine through a potential activation of proto-oncogenes and prometastatic genes (Ateeq et al., 2008). Other hypomethylating drugs and potential DNMT inhibitors have been used in (pre)clinical trials as possible therapeutics for cancer treatment. Among them, zebularine is an oral chemically stable cytidine analog lacking an amino group on position four of the pyrimidine ring with a low cellular toxicity (Yoo et al., 2004). On the other hand a next-generation hypomethylating agent named guadecitabine (SGI-110) that is a dinucleotide of decitabine linked via a phosphodiester bond to deoxyguanosine has been characterized (Griffiths et al., 2013). Guadecitabine is a prodrug of decitabine but more stable and with an extended half-life as it is resistant to cytidine deaminase as well as a prolonged clinical activity in AML and MDS (Gs et al., 2019). Further molecules including 4′-thio-2′-deoxycytidine (TdCyd) and 5-fluoro-2′-deoxycytidine (FdCyd) have been assessed in clinical trials for solid tumors and cancer treatment (Beumer et al., 2008; Thottassery et al., 2014).

**TABLE 1:** DNMT versus TET inhibitors.

Group	Subgroup	Drug	Target(s)	Effect(s)	Disease(s)	References
**DNMT inhibitors**	Nucleoside analogs	5-azacytidine (5-AZA)	DNMT1	DNA demethylation, reactivation of TSGs, leukemic cells differentiation	MDS, AML	(Bohl et al., 2018
						Kazachenka et al. (2019)
		5-aza-2′-deoxycytidine (decitabine)		DNA demethylation, cytotoxic activities	MDS, AML	Bohl et al. (2018)
		Zebularine		DNA demethylation and tumor growth inhibition, gene reexpression	Solid tumors, MDS	Yoo et al. (2004)
		Guadecitabine (SGI-110)			Haematological and solid tumors	Griffiths et al. (2013)
		4′-thio-2′-deoxycytidine (TdCyd)			Solid tumors	Thottassery et al. (2014)
		5-fluoro-2′-deoxycytidine (FdCyd)		DNA hypomethylation, cytotoxicity	Solid tumors, AML, MDS	Beumer et al. (2008)
	Non-nucleoside analogs	Procainamide	DNMT1	DNMT1 inhibition reexpression of TSGs	Solid tumors	Lee et al. (2014)
		Procaine	DNMT1, DNMT3A	DNA demethylation, apoptosis and cell proliferation inhibition		Li et al. (2018)
		Nanaomycin A	DNMT3B	DNA hypomethylation, reactivation of silenced genes	Haematological and solid tumors (*in vitro*)	Moreira-Silva et al. (2020)
		Hydralazine	DNMT1	DNA demethylation, reduction of DNMT1 activity, silenced genes reexpression, cell growth inhibition	Refractory solid tumors	
		MG98	DNMT1	DNMT1 downregulation, reexpression of hypermethylated genes	Solid tumors	Plummer et al. (2009)
		N-phthaloyl-l-tryptophan 1 (RG108)	DNMT1	DNA demethylation, reactivation of TSGs		Brueckner et al. (2005)
		Disulfiram	DNMT1	DNA demethylation, reactivation of silenced genes, growth inhibition	Refractory Multiple Myeloma and prostate cancer	Lin et al. (2011)
		SGI-1027	DNMT1, DNMT3A/B	Inhibition of DNMTs activity, induction of DNMT1 degradation, reactivation of TSGs, apoptosis	Haematological and solid tumors	Sun et al. (2018)
		epigallocatechin-3-gallate (EGCG)	DNMT1, DNMT3A/B	Inhibition of tumor proliferation, induction of cell death	Solid tumors	Chen et al. (2015)
**TET inhibitors**	Competitive inhibitors	2-Hydroxyglutarate (2-HG)	TET2	DNA hypermethylation, gene silencing, tumor progression	Haematological malignancies, AML, MDS	Ye et al. (2018)
		Fumarate	TET1/2/3	Downregulation of 5hmC levels, DNA hypermethylation		Laukka et al. (2016)
		Succinate				
	α-ketoglutarate competitive substrate	Itaconate	TET2	reduction of inflammatory responses	Haematological malignancies	Chen et al. (2022)
	Non-specific inhibitor	Dimethylallyl glycine (DMOG)	TET3	Increase of 5 mC levels, downregulation of pluripotency genes	Solid tumors	Zhang et al. (2019)
	Cytosine-based inhibitor	Bobcat339	TET1/2	Inhibition of TET activity, reduction of 5hmC levels	N/A	Chua et al. (2019)
	First-in-class TET inhibitor	C35	TET1/2/3	Inhibition of TET activity, Somatic cell reprogramming	*In vitro*	Métivier et al. (2008)
						Singh et al. (2020)
	TET-specific inhibitor	TETi76	TET1/2/3	Reduction of 5hmC levels, growth inhibition	Haematological malignancies, MDS, AML	Guan et al. (2020)

To overcome the risks of genomic and chemical instability because of using nucleoside analogs, non-nucleoside DNMTs inhibitors have been tested as antitumor drugs in the last decade (Table 1). It includes DNA binders, oligonucleotides, SAM antagonists, natural compounds as well as repurposed drugs. These small molecules may bind the catalytic site of DNMTs without incorporation into DNA, thus disrupting the interplay between DNMTs and DNA. Among non-nucleoside DNMT-inhibitors, Procainamide and its ester analog procaine are two FDA-approved drugs respectively used as anti-arrhythmic drugs and local anesthetics, which directly bind DNA and induce a decrease in DNA methylation levels in some cancers (Lee et al., 2005; Li et al., 2018). Other repurposed epigenetic inhibitors such has Nanaomycin A (DNMT3B specific inhibitor) and Hydralazine (arterial vasodilator agent) have been shown to inhibit DNA methylation, leading to genomic demethylation and TSG re-expression (Moreira-Silva et al., 2020). Nevertheless, in view of the poor specificity of these compounds, additional agents such as oligonucleotides (MG98) and SAM antagonists (N-phthaloyl-l-tryptophan 1 (RG108)) were designed to improve cancer treatments. MG98 is an antisense oligonucleotide targeting DNMT1 messenger RNA (mRNA) but, despite specifically downregulating DNMT1 in a dose-dependent manner, controversial responses have been observed in different (pre)clinical trials (Brueckner et al., 2005; Klisovic et al., 2008; Plummer et al., 2009). Similarly, Disulfiram, RG108, and the quinolone-based molecule SGI-1027 have been shown to have antitumor activities by inhibiting DNMT1 and inducing a genome-wide demethylation and TSG reactivation in cancer cells (Lin et al., 2011; Sun et al., 2018). In addition, some natural compounds including flavonoids like epigallocatechin-3-gallate (EGCG) in green tea and curcumin have also been found to have DNA demethylating properties (Chen et al., 2015). Overall, epidrugs were found especially effective to establish a more favorable epigenome for the patient when used in combination with other anticancer therapies (Yang T. et al., 2021).

Like DNMTs, UHRF1 may also be a potential effective target for cancer therapy. Indeed, besides being a regulator of DNA maintenance methylation, UHRF1 is involved in several biological processes such as embryogenesis, cell migration, proliferation as well as tumor development and cancer metastasis (Bronner et al., 2013). Numerous studies have reported that UHRF1 is highly overexpressed in many cancer types including bladder (Unoki et al., 2009), lung (Unoki et al., 2010) and colorectal carcinomas (Sabatino et al., 2012). High levels of UHRF1 in cancer cells leads to TSG silencing and DNA repair inhibition, thus contributing to tumor progression through the regulation of DNA and histone methylation. UHRF1 overexpression has also been strongly correlated with tumor aggressiveness and poor clinical outcomes. On the contrary, UHRF1 downregulation via DNA demethylation and H2 acetylation inhibits cancer development, inducing re-expression of TSGs and promoting DNA repair inhibition. UHRF1 suppression also prevents cell proliferation by inducing cell cycle arrest and apoptosis through DNA demethylation, which suggests that UHRF1 could be a potential biomarker and therapeutic target for cancer diagnosis and treatment (Ashraf et al., 2017). In this context, natural as well as chemical compounds targeting UHRF1 have been identified, but UHRF1 inhibitors have not been included yet in clinical trials (Krifa et al., 2014a, 2014b; Seo et al., 2017). Among natural compounds, uracil derivative NSC232003 (Myrianthopoulos et al., 2016), HSP90 inhibitor (Ding et al., 2016) and 4-benzylpiperidine-1-carboximidamide (BPC) (Houliston et al., 2017) have been identified by *in silico* screening. In addition, downregulation of UHRF1 and DNMT1 have also been observed with the use of natural compounds such as luteolin (Krifa et al., 2014a), EGCG (Achour et al., 2013) and hinokitiol (Seo et al., 2017). Altogether these compounds appear to be potential UHRF1 inhibitors and anti-cancer drugs but their mechanisms of action need to be further investigated.

Recently, several studies also revealed that ascorbic acid (known as Vitamin C) promotes DNA demethylation by TETs which makes it a promising therapeutic agent that could be used to increase 5 mC turnover through TET activation. Indeed, Vitamin C is a well-studied antioxidant known to work as a co-factor for Fe(II) 2-oxoglutarate dioxygenase enzymes like TET enzymes (Yin et al., 2013). By reducing ferric ions (Fe3+) to ferrous ions (Fe2+), Vitamin C enhances TET-mediated oxidation of 5 mC into 5hmC using Fe2+ and oxygen as substrates. The stimulation of TET activity promoted by Vitamin C leads to DNA demethylation and regulates gene expression by directly modulating DNA methylation and chromatin landscapes (Hore et al., 2016). Many studies reported that a large number of patients with hematological malignancies where aberrant DNA methylation is usually observed, often present Vitamin C deficiency associated with cancer progression. Higher plasma levels could be restored by oral supplementation or intravenous administration of high doses of Vitamin C and seemed to improve cancer patients outcomes, suggesting a potential role of Vitamin C as an anticancer agent (Gillberg et al., 2019). Various cancer cell lines treated with Vitamin C also presented an enhanced viral mimicry immune response to DNMTis such as decitabine (Liu et al., 2016). However, the bioavailability of Vitamin C administered orally is limited and the pharmacological doses of Vitamin C used for intravenous administration are too high for oral intake (Padayatty, 2006). Preclinical and early phase clinical trials confirmed the safety of intravenously administered Vitamin C and its efficacity in eliminating cancer cells, alone or in combination with other chemotherapies, has been tested in both hematological malignancies and solid tumors (Nauman et al., 2018). However no phase III clinical trials have been conducted yet on large cohorts of patients (Böttger et al., 2021). Studies have been carried out in mice for which oral intake of Vitamin can interfere with tumor development (Campbell et al., 2016). Recently, Cimmino et al. also reported that leukemia cells with low TET activity are more sensitive to poly (ADP-ribose) polymerase (PARP) inhibitors when TET activity is restored either genetically or pharmacologically by vitamin C (Cimmino et al., 2017). Indeed, leukemia progression was suppressed by treating AML cells with the PARP inhibitor olaparib in combination with vitamin C. Vitamin C-mediated restoration of TET2 in leukemic stem cells induces TET-mediated DNA oxidation associated DNA damage repair pathway activation. In this context, vitamin C-induced TET2 activity led to the accumulation of oxidized 5mCs and the recruitment of the BER machinery, increasing the sensitivity of leukemia cells to PARP inhibitors that block DNA repair. This suggests that vitamin C/olaparib combination is a promising therapeutic strategy for MDS and AML (Cimmino et al., 2017).

Conversely, the use of TET inhibitors to further reduce TET activity in cancer cells could also provide opportunities for cancer therapy (Table 1). Isocitrate dehydrogenases (IDH1/2), the enzymes that convert isocitrate to α-ketoglutarate in the tricarboxylic acid (TCA) cycle, are frequently mutated in many human cancer types including AML and glioblastoma (Mardis et al., 2009). These IDH1/2 mutations result in a gain of function reducing of α-ketoglutarate to 2-hydroxyglutarate (2-HG), a competitive inhibitor of αKG-dependent dioxygenases like TETs. Accumulation of the oncometabolite 2-HG inhibits TET function and contributes to tumor progression (Ye et al., 2018). Although IDH1/2 mutants exclusively produce D-(R)-enantiomer of 2-HG, the L-(S)-enantiomer 2-HG accumulation is induced in response to hypoxia and prevent TET activity. Furthermore, Fumarate and succinate are other intermediates of the TCA cycle also acting as competitive inhibitors of α-KG-dependent dioxygenases (Laukka et al., 2016). Both fumarate and succinate can decrease 5hmC levels genome-wide through inhibition of TET one and TET3. By working as a co-substrate of α-ketoglutarate, Itaconate (ITA) has also been found to be a potential TET inhibitor targeting the same catalytic site on TET2 protein and leading to TET inhibition in lipopolysaccharide (LPS)-activated macrophages, reducing inflammatory responses (Chen et al., 2022). Dimethyloxalylglycine (DMOG), a small molecule mimicking the α-ketoglutarate cofactor has also been used as a TET3 inhibitor (Zhang et al., 2019). Some studies recently reported the effect of DMOG as a TET inhibitor during embryo development by increasing 5 mC levels and downregulating pluripotency genes such as Nanog and Oct4 (Zhang J. et al., 2017; Uh et al., 2020). Nevertheless, these compounds have the potential to inhibit other α-KG-dependent dioxygenases such as histone demethylases. To design TET-specific inhibitors, cytosine-based potential inhibitors have been synthetized (Table 1) through copper-catalyzed Ullman coupling of different aromatic systems to the position five of chlorinated cytosine. In this study, Bobcat339 has been identified as the most potent TET inhibitor with mid-μM inhibiting concentrations (IC50s) for TET1 and TET2 associated with a high reduction of 5hmC levels (Chua et al., 2019). Nevertheless, Weirath et al., recently demonstrated that the inhibitory activity of Bobcat339 activity was mediated by copper contamination as no significant inhibition of human TET1 and TET2 enzymes was observed. *In silico* screening of natural products as potential TET inhibitors further identified compound 35 (C35), a catechol containing small molecule targeting the catalytic core of TETs and thus blocking their enzymatic activity (Singh et al., 2020). While several putative TET inhibitors have been identified, further investigations and clinical studies are required to assess their pharmacological efficacy *in vivo*. However, based on the observation that IDH and TET mutations are likely to be synthetic lethal, TET inhibition with the synthetic compound TETi76 in aTET2 mutant context was shown to suppress clonal evolution and growth of human leukemia xenografts (Guan et al., 2021). These data demonstrate that induction or reduction of TET activity in cancer cells both provide interesting opportunities for establishing new and context-dependent anti-cancer strategies.

Given that changes in DNA methylation landscapes occur through aging, epigenetic clocks have been designed, using DNA methylation levels from a set of CpGs in the genome, to measure and predict biological aging from human tissues (Hannum et al., 2013; Horvath, 2013). These biological age predictors use trained algorithms and the occurrence of age-related lifespan as biomarkers (Levine et al., 2018; Lu et al., 2019; McEwen et al., 2020; De Lima Camillo et al., 2022). While chronological age is defined as the time an individual has been alive since birth, biological age refers to the physiological age by considering health, age-associated diseases, morbidity, mortality as well as lifestyle factors and DNA methylation (Porter et al., 2021). For instance, two individuals with the same chronological age can have different biological ages which are strongly associated with DNA methylation levels. Given that changes in DNA methylation landscapes occur through aging, epigenetic clocks have been designed, using DNA methylation levels from a set of CpGs in the genome, to measure and predict biological aging from human tissues (Hannum et al., 2013; Horvath, 2013). These biological age predictors use trained algorithms and the occurrence of age-related lifespan as biomarkers (Levine et al., 2018; Lu et al., 2019; McEwen et al., 2020; De Lima Camillo et al., 2022). While chronological age is defined as the time an individual has been alive since birth, biological age refers to the physiological age taking into account health, age-associated diseases, morbidity, mortality as well as lifestyle factors and DNA methylation (Porter et al., 2021). Hence, two individuals with the same chronological age can have different biological ages in association with different DNA methylation levels. In the context of B cell malignancies, the proliferative history of the cells strongly impacts on DNA methylation levels, especially in the late-replicating heterochromatin (Duran-Ferrer et al., 2020). Based on these observations, the group of I. Martin-Subero developed a new epigenetic clock called epiCMIT that shows strong correlation with proliferative history independently of chronological age and is an efficient predictor of the clinical behavior in B-cell tumors (Duran-Ferrer et al., 2020). The discovery that DNA methylation-based epigenetic clocks could be used as diagnostic tools and age prediction biomarkers reinforced the idea that understanding DNA methylation dynamics in disease is relevant for human cancer treatment.

The regenerative capacities of cells and tissues are gradually lost over time as people age, making them become more vulnerable to age-related diseases. The breakthrough discovery of induced pluripotent stem cell (iPSC) reprogramming by Yamanaka et al., in 2006, allowed to generate pluripotent cells from differentiated somatic cells (Takahashi and Yamanaka, 2006). Indeed, by overexpressing four pluripotency TFs (Oct4, Sox2, Klf4 and c-Myc) known as Yamanaka factors, almost any non-dividing, differentiated somatic cell can be reprogrammed to a stem-cell like state through a dedifferentiation process, thus reversing many age-related changes, including the epigenetic clock. iPSCs reprogramming appears to be a promising technology in regenerative medicine to treat many neurodegenerative diseases by producing patient-specific iPSCs which could minimize incompatibility. As dedifferentiation also occurs in cancer (Friedmann-Morvinski and Verma, 2014) and iPSC reprogramming causes loss of cell identity and function, other reprogramming based strategies have emerged to rejuvenate aging cells and tissues. Gill et al., recently developed the first “maturation phase transient reprogramming” (MPTR) method to rejuvenate the epigenome of cells (Gill et al., 2022). This *in vitro* reprogramming system is based on the ectopic expression of Yamanaka factors in mouse fibroblasts when the mesenchymal-epithelial-transition phase of reprogramming is reached, and then their expression is abolished. Using multi-omics approach and epigenetic clocks, MPTR has been found to rejuvenate several hallmarks of aging by reducing both transcriptional and epigenetic ages by 30 years and reverse age-associated changes without losing cell identity. These observations have been recently supported by *in vivo* transient reprogramming studies (Chondronasiou et al., 2022). Altogether, these findings demonstrate that pluripotency reprogramming and the rejuvenation program can be dissociated. Therefore, these strategies could smooth the way for the identification of potential new anti-ageing therapeutic targets with the ability to promote rejuvenation.

## Conclusion

For the last decades, emerging high-throughput technological advances and mathematical modelling largely contributed to unravel DNA methylation oscillations at both local and genome scales. DNA methylation has been highlighted as a dynamic cycling process regulated by a balance between DNMTs and TETs and associated with a constant turnover of cytosine modifications. These variable steady state DNA methylation levels generate heterogenous DNA methylation patterns in healthy cells but also in cancer cells. Driven by DNA methylation dynamics, tumor heterogeneity can act as a barrier in cancer treatment by supporting tumor cells progression. Within the last years, DNA methylation has been increasingly investigated as a potential biomarker and therapeutic target for human cancer treatment. DNA methylation-based epigenetic clocks have been developed as diagnostic tools and age prediction biomarkers, while transient reprogramming strategies reinforced our comprehension of DNA methylation dynamics to design new therapies. Several molecules targeting methylating (DNMTs) and demethylating (TETs) enzymes were identified as potential anticancer drugs through modulations of DNA methylation dynamics. While some epidrugs are currently used alone or in combination with other therapies for cancer treatment, one of the main challenges remains to reduce side effects and cytotoxicity to ensure a positive outcome for patients.
